# Impact and management of drooling in children with neurological disorders: an Italian Delphi consensus

**DOI:** 10.1186/s13052-022-01312-8

**Published:** 2022-07-19

**Authors:** Antonella Riva, Elisabetta Amadori, Maria Stella Vari, Alberto Spalice, Vincenzo Belcastro, Maurizio Viri, Donatella Capodiferro, Antonino Romeo, Alberto Verrotti, the Delphi panel experts’ group, Maria Francesca Aiello, Irene Bagnasco, Pier Antonio Battistella, Stefania Bergamoni, Benedetta Boldrini, Pasquale Bratta, Andrea Brusaferro, Mario Brusco, Beatrice Burchiani, Elisa Burdino, Beatrice Cardinali, Morena Cassani, Elena Cavalli, Anna Cavallini, Maria Cordelli Duccio, Gaetano D’agata, Giovanna Di Corcia, Gianluca D’onofrio, Giulia Fagiolari, Antonella Fattorusso, Matteo Felicioni, Federica Gaiotti, Cristina Galati, Luisa Gasola, Giuseppina Giaquinto, Chiara Gizzi, Domenico Leonardo Grasso, Chiara Isidori, Maria Teresa Marcucci, Valentina Mazzoni, Elisabetta Mencaroni, Gianluca Monacelli, Francesco Nicita, Alessandro Orsini, Annamaria Pellegrino, Cinzia Peruzzi, Gianluca Piccolo, Ilaria Pistola, Giovanni Prezioso, Patrizia Pulitano, Vincenzo Raieli, Marina Saladino, Annamaria Sapuppo, Rossella Sica, Carlotta Spagnoli, Maria Tagliente, Giorgia Tascini, Gaetano Terrone, Eleonora Tulli, Valerio Vinti, Pasquale Striano

**Affiliations:** 1 Pediatric Neurology and Muscular Diseases Unit, IRCCS Istituto Giannina Gaslini, Via Gaslini 5, 16148 Genoa, Italy; 2 Department of Neurosciences, Rehabilitation, Ophthalmology, Genetics, Maternal and Child Health, University of Genova, Genoa, Italy; 3 Department of Pediatrics, “Sapienza” University of Rome, Rome, Italy; 4 Neurology Unit, Maggiore Hospital, Lodi, Italy; 5 Department of Child Neuropsychiatry, Hospital Maggiore della Carità, Novara, Italy; 6 Department of Biomedical Sciences and Human Oncology, Neonatology and Intensive Care Neonatal Unit Section, University of Bari Aldo Moro, Bari, Italy; 7 Pediatric Neurology Unit and Epilepsy Center, Fatebenefratelli Hospital, ASST Fatebenefratelli Sacco, Milan, Italy; 8 Department of Pediatrics, University of Perugia, Perugia, Italy

**Keywords:** Drooling, Neurological disorders, Pediatrics, Cerebral palsy, Delphi

## Abstract

**Background:**

The rate of chronic drooling in children older than 4 years is 0.5%, but it rises to 60% in those with neurological disorders. Physical and psychosocial consequences lead to a reduction in the quality of Life (QoL) of affected patients; however, the problem remains under-recognized and under-treated. We conducted an Italian consensus through a modified Delphi survey to discuss the current treatment paradigm of drooling in pediatric patients with neurological disorders.

**Methods:**

After reviewing the literature, a board of 10 experts defined some statements to be administered to a multidisciplinary panel through an online encrypted platform. The answers to the questions were based on a 1–5 Likert scale (1 = strongly disagree; 5 = strongly agree). The scores were grouped into 1–2 (disagreement) and 4–5 (agreement), while 3 was discarded. The consensus was reached when the sum of the disagreement or agreement was ≥75%.

**Results:**

Fifteen statements covered three main topics, namely clinical manifestations and QoL, quantification of drooling, and treatment strategies. All statements reached consensus (≥75% agreement). The 55 Italian experts agreed that drooling should be assessed in all children with complex needs, having a major impact on the QoL. Attention should be paid to investigating posterior hypersalivation, which is often neglected but may lead to important clinical consequences. Given that the severity of drooling fluctuates over time, its management should be guided by the patients’ current needs. Furthermore, the relative lack of validated and universal scales for drooling quantification limits the evaluation of the response to treatment. Finally, the shared therapeutic paradigm is progressive, with conservative treatments preceding the pharmacological ones and reserving surgery only for selected cases.

**Conclusion:**

This study demonstrates the pivotal importance of a multidisciplinary approach to the management of drooling. National experts agree that progressive treatment can reduce the incidence of complications, improve the QoL of patients and caregivers, and save healthcare resources. Finally, this study highlights how the therapeutic strategy should be reconsidered over time according to the available drugs on the market, the progression of symptoms, and the patients’ needs.

**Supplementary Information:**

The online version contains supplementary material available at 10.1186/s13052-022-01312-8.

## Background

Drooling, or excessive salivation, is a normal condition in infants, but it usually stops between 15 and 18 months of age. The presence of chronic drooling in children older than 4 years is considered pathologic [1–3]. Overall, the reported prevalence of chronic drooling is approximately 0.5% in the pediatric population, but this figure rises to 60% in children affected by neurological disorders, such as cerebral palsy, or, more rarely, Dravet syndrome, Rett syndrome, Goldenhar and Angelman syndromes [3–5]. Dysfunction in the oral–motor control is the most important predisposing factor to drooling; other causes include dental malocclusion and hypersalivation [4]. Drooling could either be anterior – and therefore identifiable at the clinical examination – or posterior. The latter is usually not easily identified but is equally troublesome for patients.

Indeed, drooling can have physical and psychological consequences for the affected patients. Physical complications can include oral maceration, leading to secondary infection, dehydration, speech difficulties and bad breath, while psychosocial consequences include a sense of isolation and increased dependency on caregivers [1, 6, 7]. These consequences lead to a marked reduction in the quality of life (QoL) of the affected patients [8]. Despite these major consequences, drooling remains an under-recognized symptom in children with neurological disorders. The correct diagnostic and therapeutic approaches to this condition require further investigations [2, 3, 5]. In such a scenario, due to the lack of firm evidence, a major role is acquired by experts’ opinion, which can be addressed through consensus approaches, such as the Delphi Panel, a methodology increasingly used also in the field of pediatrics [9–13].

We have conducted a modified Delphi process with experienced Italian pediatricians and pediatric neurologists, with the aim to discuss the current management of drooling in pediatric patients with neurological disorders.

## Methods

**Fig. 1:**
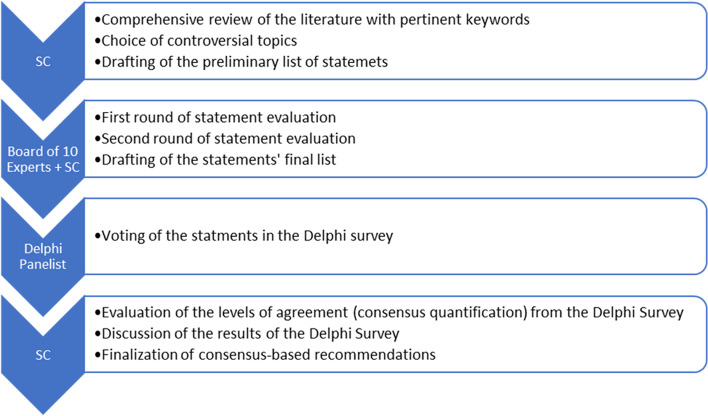
Project workflow*.* SC: steering committee

An overview of the study methodology is provided in Fig. 1. Firstly, in December 2020, a Steering Committee carried out a comprehensive review of the literature using multiple combinations of pertinent keywords. The Steering Committee comprised three authors with experience in treating drooling in pediatric patients with neurological disorders. The Steering Committee then assembled at an online meeting and selected three controversial topics concerning drooling in pediatric patients with neurological disorders. Selected topics were (i) clinical manifestations and impact on the QoL; (ii) quantification; and (iii) treatment strategies. Finally, the Steering Committee defined a series of statements regarding these topics. These statements were then evaluated, discussed, and revised by an enlarged board of 10 experts composed of pediatricians, pediatric neurologists and rehabilitation specialists through two rounds of evaluation. In each round, statements could be modified, merged or removed, and other statements could be added until a final list was defined.

Once statements had been finalized, the final list of statements was administered to a multidisciplinary panel of 55 Italian experts (see Supplementary file Table 1) through the Delphi Method. The Delphi Method is a well-established method of consensus which takes place in an interactive and anonymous way, often through online surveys, with a group of appropriately selected experts. Several rounds or phases of evaluation and expression of opinions of the experts are used to validate statements through a Likert scale (1 = strongly disagree, 2 = disagree, 3 = neither disagree nor agree, 4 = agree and 5 = strongly agree), thus providing a highly structured and transparent process to obtain feedback [11, 14–16]. The aims of the Delphi survey are to evaluate the level of agreement (consensus quantification) and to resolve differences of opinion (consensus development).

Our Delphi round was conducted in March and April 2021. All panel members were chosen based on their curricula and had proved expertise in the management of drooling in children. To provide a multidisciplinary approach and to ensure a homogenous territorial distribution, members from different backgrounds and discipline – namely pediatrics, neurology, dentistry, and rehabilitation – as well as from different geographical areas were chosen. Panel members were asked to fill in an online questionnaire through a secure web platform. The answers were collected anonymously to reduce the risk of bias or influence by other specialists’ opinions.

The absolute number and percentage of participants who scored each item as 1–2 (disagreement) or 4–5 (agreement) was then calculated. The number of participants who scored items as 3 was discarded, since it did not indicate either agreement or disagreement. The consensus on agreement was reached when the percentage of panelist voting 4 and 5 was ≥75%. The consensus on disagreement was considered to be reached when the percentage of panelist voting 1 and 2 was ≥75%.

The Steering Committee discussed the results of the Delphi survey, and a series of consensus-based recommendations were finalized.

## Results

The Steering Committee elaborated a preliminary list of 28 statements. After the two-round discussion with the enlarged board, some statements were merged or removed. The final list consisted of 15 statements regarding the three identified controversial topics: clinical manifestation and impact on the QoL, quantification, and treatment strategies. The original, intermediate, and final list of statements can be found in the Supplementary file Table 2.

Table 1 depicts the results of the Delphi survey for every final statement. All the 15 statements reached consensus on agreement.

**Table 1:** Statements and results of the voting. All statements reached consensus on agreement (i.e., sum of 5 + 4 ≥ 75%)

Topic	Statement	Level of agreement (% of all voters, *n* = 55)						Consensus
		*5*	*4*	*3*	*2*	*1*	*Sum 5 + 4*	
Clinical manifestations and QoL	Drooling is one of the symptoms that I often evaluate in patients with complex disabilities	50	46	4	0	0	96	Reached, agreement
	Drooling is often a clinically relevant symptom in at least half of patients with infantile cerebral palsy	91	9	0	0	0	100	Reached, agreement
	Drooling is a frequent symptom of some rare pediatric diseases	80	20	0	0	0	100	Reached, agreement
	Drooling severity can vary over time	85	15	0	0	0	100	Reached, agreement
	Drooling leads to a reduction in the QoL of the patient and those who take care of it	98	2	0	0	0	100	Reached, agreement
	It is useful to evaluate drooling symptom in all patients with chronic neurological diseases	86	14	0	0	0	100	Reached, agreement
Quantification	The assessment of drooling severity must be monitored over time with quantitative scales	69	24	7	0	0	93	Reached, agreement
	Physicians must record the severity of drooling in the medical records	78	22	0	0	0	100	Reached, agreement
	It is important to distinguish between anterior and posterior hypersalivation	84	13	4	0	0	97	Reached, agreement
Treatment strategies	Drooling therapies are possibly prescribed only by the child neuropsychiatrist, neurologist, pediatrician	43	39	18	0	0	82	Reached, agreement
	Rehabilitation therapy must precede pharmacological therapy and surgical options	80	18	2	0	0	98	Reached, agreement
	Non-invasive drug therapy (e.g., oral use) must always precede invasive therapy (e.g., botulinum toxin)	78	18	4	0	0	96	Reached, agreement
	Pharmacological therapy of drooling is essentially based on the use of products that have no specific indication (e.g., antihistamines)	27	48	25	0	0	75	Reached, agreement
	Botulinum toxin A is administered to pediatric patients only in a hospital setting, after sedation, and with ultrasound control	88	9	4	0	0	97	Reached, agreement
	During the entire period of action of botulinum toxin A, no other drugs are given to control drooling	39	41	20	0	0	80	Reached, agreement

*QoL* Quality of life

## Discussion

Despite being associated with important physical and psychological consequences, drooling is still an under-recognized and under-treated symptom in pediatric patients with neurological diseases, and the most correct approach to its management in clinical practice remains undefined [2, 3]. In particular, the most suitable assessment methods and the correct therapeutic strategy need clarifications since studies on these topics remain scant [3].

Given the lack of well-grounded evidence on drooling management, we have conducted a Delphi panel process involving several Italian experts from different disciplines (pediatrics, neurology, dentistry, rehabilitation), with the aim to discuss the current management of drooling in pediatric patients with neurological disorders.

The outcome of the consensus process consisted of 15 statements, all approved by the Delphi panel, and concerning three main areas: clinical manifestation and impact on the QoL, quantification and treatment strategies.

### Clinical manifestations

Drooling remains a neglected symptom in clinical practice, despite being highly prevalent, particularly in those children with cerebral palsy or rare neurological disorders [2, 4]. Indeed, this symptom is rarely assessed in daily practice, and the panel agreed that it should be methodically evaluated in all children with complex needs. Furthermore, although anterior drooling is the most obvious and disabling, the concomitant or isolated presence of posterior drooling is seldom assessed. Indeed, as posterior drooling is very frequent in neurological patients [3] and potentially dangerous for patients’ health (e.g., aspiration pneumonia, chronic respiratory disorders), it should also be evaluated in the clinical setting through an anamnestic interview of the caregivers.

Remarkably, all the Italian experts agreed that drooling may fluctuate in their patients over time; a point already highlighted by Speyer and his collaborators [5] even emphasized the relevance of strictly monitoring it over time it implies changes in the therapeutic paradigm.

### Impact on QoL

Drooling has a major impact on the QoL of the affected patients, who may end up feeling a sense of isolation and rejection by peers [8]. Furthermore, the presence of drooling poses a significant additional burden to the caregivers of the affected patients, who often experience difficulties in managing and dealing with the underlying neurological disorder of their child [3, 7]. However, caregivers often do not report the presence of drooling to the treating physicians [5] and should therefore be encouraged to do so to allow prompt management of this symptom, with ultimate benefits on the overall quality of care.

### Quantification

The Delphi panel agreed on the need to accurately assess the severity of drooling in all children with neurological conditions using validated scales and recording the assessment results in the clinical chart. Assessment should be performed frequently over time to identify any change in the severity of drooling and redefine the management strategy accordingly.

However, at present, only a few scales to assess drooling are available, and the three most commonly used subjective scales (Drooling Impact Scale, modified Teachers’ Drooling Scale, and Drooling Severity and Frequency Scale) are still scantly studied and validated in daily practice [3, 4, 17, 18]. On the other hand, quantitative scales provide a more reliable evaluation of the severity of drooling but may be difficult in clinical practice [3]. These quantitative scales include Sochaniwskyj’s technique, the Drooling Quotient, and gland scintigraphy [19–22], which uses radioactive isotopes. However, this latter method is quite invasive, and therefore its application in daily practice is largely limited, particularly in pediatrics.

The goal of the upcoming years is the development of validated and quantitative scales to easily measure the severity of drooling in everyday life, allowing a more careful diagnosis and monitoring of the evolution of this symptom. An accurate scale should investigate the severity and frequency of drooling, as well as all its physical and psychological complications.

### Treatment strategies

According to the Delphi panel, multidisciplinary management of drooling is of great importance. A comprehensive evaluation of the available treatments for drooling goes beyond the scopes of the present manuscript and can be found elsewhere [3]; although, it is clear that treatment should follow a progressive pattern, with rehabilitation and conservative approaches preceding pharmacological treatment. Conservative approaches include behavioral and bio-functional therapies, physiotherapy, and biofeedback technique [1, 23]. Each of the previous approach is primarily intended to make children aware of drooling through various means, such as mirrors (Hussein 1998 [23]), specific patients-designed appliances (i.e., mouth vestibular braces and stimulating palatal plates) [23, 24], or time-fixed auditory signals [23, 25]. However, these methods require high collaboration, and are primarily addressed to children older than 8 years with mild up to moderate intellectual disability. Moreover, patients usually become familiar with the stimulus at long-term follow-up.

When medical therapy becomes necessary, less invasive therapies (e.g., those orally administered) should be preferred in the first-line over more invasive ones, such as Botulinum toxin, which requires multiple inpatient administration under sedation. Some randomized clinical trials using either toxin A or toxin B against placebo have been conducted in adult patients with chronic drooling mainly caused by Parkinson’s disease [26, 27]. However, the effects commonly last for 6 weeks up to 6 months, and multiple injection should be required during time, with a high likelihood of effect loss and immunogenic effects in the younger [28]. Finally, surgery should be saved for more severe cases when all other approaches have failed [3].

This progressive treatment approach may reduce the incidence of complications, improve the QoL for the patients and their caregivers, and save healthcare resources. In this perspective, at the Italian level, experts agreed that no additional drugs are needed during the effective period of action of botulinum toxin A. However, the treatment strategy should always be reconsidered over time and, given that botulinum is not a “definitive” treatment, combined multitherapy may become necessary.

It is worth noticing that drugs with a specific indication for drooling (glycopirronium for oral administration; Botulinum toxin for injection, with administration pending approval according to 648 Law in Italy) have only recently entered the market; hence, clinicians are still largely using products that have no specific indication. In the next few years, this would change with more specific drugs gaining the market. In particular, glycopirronium proved to be more effective in treating drooling than other molecules requiring oral administration [3, 21].

## Conclusion

This study suggests how a multidisciplinary approach to the management of drooling is of utmost importance. At the national level, experts agree that progressive treatment can reduce the incidence of complications, improve the QoL of patients and caregivers, and save healthcare resources due to treatments prescription. Finally, this study highlights how the therapeutic strategy should be reconsidered over time according to the entering of new and more specific drugs on the market, particularly to the progression of symptoms and the specific needs of patients and their care providers.

## Supplementary Information

**Additional file 1: Supplementary file Table 1.** The Delphi Panel members.

**Additional file 2: Supplementary file Table 2.** Statement changes during the statement evaluation process.

## Data Availability

All data will be available upon reasonable request.

## Abbreviations

QoL: quality of life
