# Supplementary material for: Impact and management of drooling in children with neurological disorders: an Italian Delphi consensus
Source: Ital J Pediatr. 2022 Jul 19;48:118. doi: 10.1186/s13052-022-01312-8 (PMC9297577; doi:10.1186/s13052-022-01312-8)
Supplement: Supplementary file 1 — Additional file 1: Supplementary file Table 1. The Delphi Panel members. [file 13052_2022_1312_MOESM1_ESM.docx]

**Supplementary file Table 1** – The Delphi Panel members

| **Name** | **Surname** |
| --- | --- |
| Maria Francesca | Aiello |
| Irene | Bagnasco |
| Pier Antonio | Battistella |
| Stefania | Bergamoni |
| Benedetta | Boldrini |
| Pasquale | Bratta |
| Andrea | Brusaferro |
| Mario | Brusco |
| Beatrice | Burchiani |
| Elisa | Burdino |
| Beatrice | Cardinali |
| Morena | Cassani |
| Elena | Cavalli |
| Anna | Cavallini |
| Maria | Cordelli Duccio |
| Gaetano | D'agata |
| Giovanna | Di Corcia |
| Gianluca | D'onofrio |
| Giulia | Fagiolari |
| Antonella | Fattorusso |
| Matteo | Felicioni |
| Federica | Gaiotti |
| Cristina | Galati |
| Luisa | Gasola |
| Giuseppina | Giaquinto |
| Chiara | Gizzi |
| Domenico Leonardo | Grasso |
| Chiara | Isidori |
| Maria Teresa | Marcucci |
| Valentina | Mazzoni |
| Elisabetta | Mencaroni |
| Gianluca | Monacelli |
| Francesco | Nicita |
| Alessandro | Orsini |
| Annamaria | Pellegrino |
| Cinzia | Peruzzi |
| Gianluca | Piccolo |
| Ilaria | Pistola |
| Giovanni | Prezioso |
| Patrizia | Pulitano |
| Vincenzo | Raieli |
| Antonella | Riva |
| Antonino | Romeo |
| Marina | Saladino |
| Annamaria | Sapuppo |
| Rossella | Sica |
| Carlotta | Spagnoli |
| Pasquale | Striano |
| Maria | Tagliente |
| Giorgia | Tascini |
| Gaetano | Terrone |
| Eleonora | Tulli |
| Maria Stella | Vari |
| Alberto | Verrotti |
| Valerio | Vinti |
