# Supplementary material for: Impact and management of drooling in children with neurological disorders: an Italian Delphi consensus
Source: Ital J Pediatr. 2022 Jul 19;48:118. doi: 10.1186/s13052-022-01312-8 (PMC9297577; doi:10.1186/s13052-022-01312-8)
Supplement: Supplementary file 2 — Additional file 2: Supplementary file Table 2. Statement changes during the statement evaluation process. [file 13052_2022_1312_MOESM2_ESM.docx]

| **Supplementary file Table 2** – Statement changes during the statement evaluation process | | |
| --- | --- | --- |
| **Original statements** | **Statements after the first evaluation** | **Statements after the second evaluation** |
| Drooling is a frequent symptom in pediatric patients with chronic neurological disorders | Drooling is a symptom that I methodically evaluate in patients with complex disabilities | Drooling is one of the symptoms that I often evaluate in patients with complex disabilities |
| Most patients with drooling are diagnosed with infantile cerebral palsy | *Unchanged* | Drooling is often a clinically relevant symptom in at least half of patients with infantile cerebral palsy |
| In rare pediatric diseases such as Angelman, Rett, Goldenhar, it is not uncommon to find Drooling | Drooling is a frequent symptom of some rare pediatric diseases | *Unchanged* |
| The severity of drooling in pediatric patients is not constant over time | The severity of drooling in pediatric patients fluctuates over time | Drooling severity can vary over time |
| Drooling in pediatric patients leads to a reduction in the quality of life of the patient and those who take care of it | *Unchanged* | Drooling leads to a reduction in the QoL of the patient and those who take care of it |
| Drooling in pediatric patients is a cause of damage to instruments used by the patient (notebooks, books, pcs, etc.) | *Removed* |  |
| Drooling in pediatric patients leads to social isolation and reduced self-esteem | *Unchanged* | *Removed* |
| The pediatric neuropsychiatrist, neurologist, or pediatrician is required to evaluate the drooling symptom in all pediatric patients with chronic neurological diseases | It is useful for the pediatric neuropsychiatrist, neurologist, and pediatrician doctor to evaluate the drooling symptom in all pediatric patients with chronic neurological diseases | It is useful to evaluate the drooling symptom in all patients with chronic neurological diseases |
| The assessment of drooling severity in pediatric patients must be performed by the doctor with quantitative scales; patients should also be monitored over time by the caregiver, continuing with the same scales used. | *Unchanged* | The assessment of drooling severity must be monitored over time with quantitative scales |
| The pediatric neuropsychiatrist or pediatric neurologist reports the data of drooling severity in the pediatric patient’s medical record in order to be able to monitor its evolution | *Unchanged* | Physicians must record the severity of drooling in the medical records |
| Drooling in pediatric patients is defined as severe when saliva drips from the chin/mouth onto clothing | The severity of drooling in pediatric patients must always be assessed with appropriate scales | *Removed* |
| In the evaluation of drooling in pediatric patients, it is important for the doctor to evaluate whether it is anterior or posterior drooling due to the different entailing medical implications | It is important to evaluate/distinguish between anterior and posterior drooling | It is important to distinguish between anterior and posterior drooling |
| The diagnosis of drooling in pediatric patients is carried out by the child psychiatrist, neurologist, pediatrician | *Removed* |  |
| The therapy of drooling in pediatric patients is prescribed by the pediatric neuropsychiatrist, neurologist, pediatrician | The therapies of drooling in pediatric patients are prescribed by the pediatric neuropsychiatrist, neurologist, pediatrician | Drooling therapies are possibly prescribed only by the child neuropsychiatrist, neurologist, pediatrician |
| The therapy of drooling in pediatric patients can be: rehabilitative, pharmacological, surgical. | *Removed* |  |
| Rehabilitation therapy must always precede pharmacological therapy and surgical options | *Unchanged* | Rehabilitation therapy must precede pharmacological therapy and surgical options |
| Rehabilitation therapy must always precede surgical options | *Removed* |  |
| In pharmacologic therapies in pediatric patients, non-invasive therapy (e.g., oral use) must precede invasive drug therapy (e.g., infiltrations) | *Unchanged* | Non-invasive drug therapy (e.g., oral use) must always precede invasive therapy (e.g., botulinum toxin) |
| Non-invasive drug therapy in pediatric patients is essentially based on the use of products that have no specific indication | The therapy of drooling in pediatric patients is essentially based on the use of products that have no specific indication | Pharmacological therapy of drooling is essentially based on the use of products that have no specific indication (e.g., antihistamines) |
| In non-invasive drug therapy, a drug with a specific indication for drooling in pediatric patients should be used before products that do not have a specific indication | *Removed* |  |
| In non-invasive drug therapies of drooling in pediatric patients, drugs with fewer central side effects should be preferred | *Removed* |  |
| Invasive drug therapy in pediatric patients must precede the surgical option | *Removed* |  |
| Infiltrations of botulinum toxin A in the salivary glands are therapeutic options suitable only for children/adolescents with drooling unresponsive to non-invasive therapies | *Unchanged* | *Removed* |
| Botulinum toxin A is administered to pediatric patients only in a hospital setting, after sedation, and with ultrasound control | *Unchanged* | *Unchanged* |
| The dose of botulinum toxin A administered to pediatric patients controls the severe drooling symptom for 4 months on average | *Removed* |  |
| During the entire period of action of botulinum toxin A in pediatric patients, other drugs are also administered to control drooling | *Unchanged* | During the entire period of action of botulinum toxin A, no other drugs are administered to control drooling |
| In the pediatric patient with severe drooling, the infiltration of botulinum toxin A can be repeated even in the short term | *Removed* |  |
| In the pediatric patient with severe drooling, the infiltration of botulinum toxin A can be repeated throughout childhood and adolescence | *Removed* |  |
